# Reducing All-cause 30-day Hospital Readmissions for Patients Presenting with Acute Heart Failure Exacerbations: A Quality Improvement Initiative

**DOI:** 10.7759/cureus.7420

**Published:** 2020-03-25

**Authors:** Raunak Nair, Hassan Lak, Seba Hasan, Deepthi Gunasekaran, Arslan Babar, K V Gopalakrishna

**Affiliations:** 1 Internal Medicine, Cleveland Clinic - Fairview Hospital, Cleveland, USA; 2 Internal Medicine, Cleveland Clinic Foundation, Cleveland, USA

**Keywords:** chf, readmission, heart failure, quality improvement, patient education, follow-up appoitment

## Abstract

Background

Congestive heart failure (CHF) is the most common cause of hospitalization in the US for people older than 65 years of age. It has the highest 30-day re-hospitalization rate among medical and surgical conditions, accounting for up to 26.9% of the total readmission rates. We conducted a quality improvement project at our hospital with the objective to reduce the 30-day all-cause readmissions of patients with CHF by improving the transition of care and setting up scheduled follow-up appointments within two weeks of patient discharge.

Method

Retrospective data were collected to understand the pattern of admission for CHF during November 2017. Data on 30-day readmission post-discharge was also collected to understand readmission rates. Similarly, all patients who were admitted with acute CHF exacerbation to our hospital during the month of November 2018 were included in our intervention cohort. The 30-day readmission rates of these patients post-intervention were calculated and compared to the initial cohort.

Results

As part of our study, we ensured that 58% of the enrolled patients had a follow-up appointment scheduled within two weeks of discharge compared to only 30% in 2017. Also, 56% of the enrolled patients kept their follow-up appointments compared to 37% in 2017. The 30-day readmission rate of CHF patients was reduced in half after the implementation of our project, with a 14% readmission rate for our study patients compared to 28% in 2017.

Conclusion

Patient education and measures to augment post-discharge follow-up appointments can lead to substantial reductions in the readmission rates of heart failure (HF) patients.

## Introduction

Heart failure (HF) is a chronic disorder that affects around 5.7 million people annually in the US and contributes to an annual expenditure of around $30.7 billion [1]. The total medical spending on HF is expected to rise to around $53 billion by 2030 [2]. HF also contributes to significant mortality, with only 30-40% of patients surviving up to one year after being hospitalized for HF [3,4]. The impact of HF also extends to the post-discharge course. The 30-day readmission rate for HF has been reported to be around 23% [5]. Such readmissions not only impose significant distress on the patients but also increase the burden on healthcare. To reduce the number of preventable readmissions, the Centers for Medicare & Medicaid Services (CMS) initiated the Hospital Readmissions Reduction Program (HRRP) in 2012. As per the HRRP, CMS will reduce payments to hospitals with higher than expected readmission rates following admissions with HF [6]. Though the risk-adjusted readmission rates have started to decline after the initiation of HRRP, the readmission rates in several hospitals still remain high [7].

Solutions for Value Enhancement (SolVE) cohort is a quality improvement training program established by the Cleveland Clinic's Graduate Medical Education program to promote excellence in medical education [8]. The duration of this program is 12 weeks, and it aims to identify interventions that can lead to better patient care and to train caregivers to successfully lead quality improvement initiatives. Our SolVE team identified that the readmission rate of HF patients in our hospital was higher than the national average and that there was definitely room for improvement. Since these readmissions contribute to significant morbidity and mortality, we aimed to find interventions that could help us reduce our institution's readmission rate by at least 25% in 8-12 weeks.

## Materials and methods

Our project involved retrospective data collection of all patients with a primary diagnosis of congestive heart failure (CHF) who were admitted to our hospital during the month of November in 2017. Readmission rates were calculated as the percentage of CHF patients who were readmitted to our hospital within 30 days of discharge for any unplanned cause. All data were collected from electronic medical records. These patients acted as our control group. Similarly, all patients who were admitted to our hospital with CHF exacerbation during November 2018 were included in our study group. Patients who were younger than 18 years old, who were on dialysis, and those with discharge disposition other than their homes were excluded from both the control and study groups.

In the study group, patient education materials regarding CHF exacerbation, lifestyle modifications, the importance of medication compliance, and the importance of follow-ups were provided. Instructions were also given to all nurses taking care of these patients to educate the patients before discharge. Additionally, all nursing staff and health unit coordinators (HUC) were asked to ensure that follow-up appointments were scheduled with patients' primary care physician (PCP) and cardiologist or the HF clinic before they were discharged.

## Results

Pre-intervention scenario

**Figure 1:**
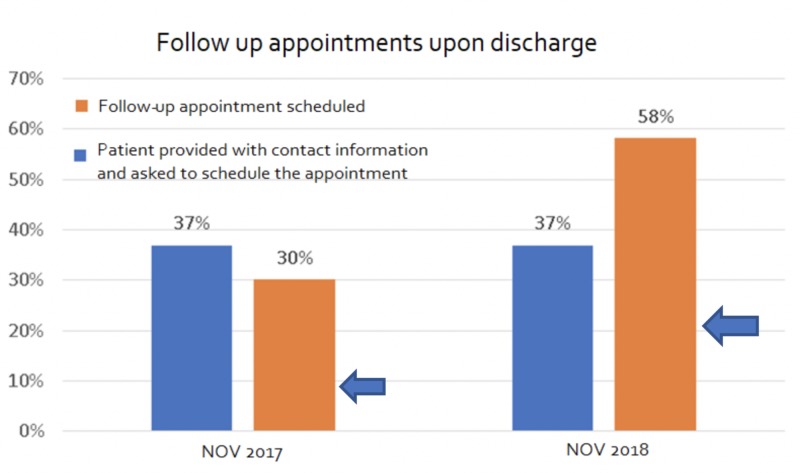
Comparison of follow-up appointments since discharge between the control and intervention groups NOV 2017 represents the control group and NOV 2018 represents the intervention group

**Figure 2:**
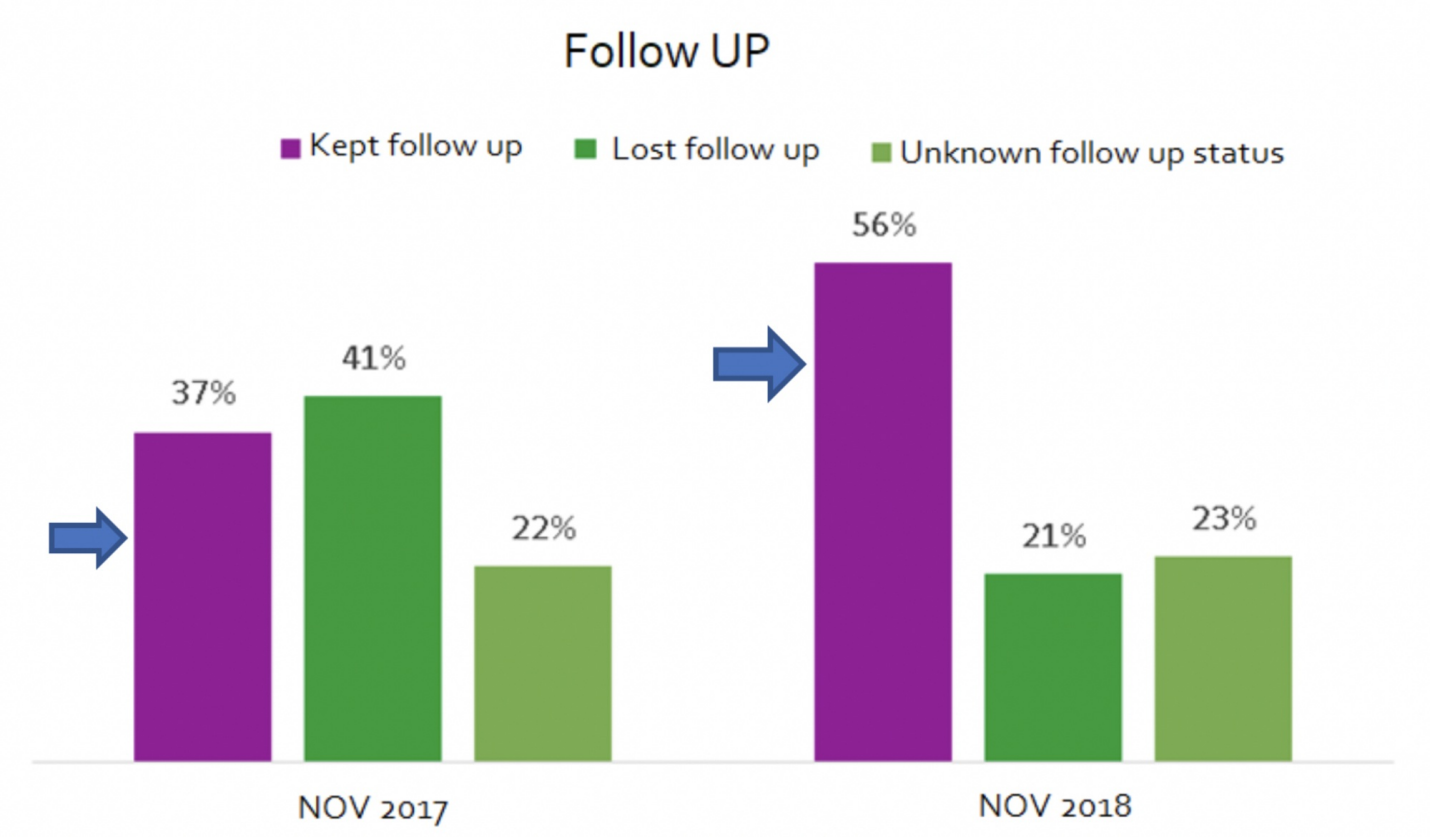
Comparison of successful follow-up appointments between the control and intervention groups NOV 2017 represents the control group and NOV 2018 represents the intervention group

Before the implementation of our project, we found that the all-cause readmission rate of patients admitted with CHF to our hospital was around 28%. Only 30% of all the patients with CHF had a scheduled follow-up appointment with the PCP or cardiologist on discharge (Figure 1). Of all the patients who were discharged, only 37% kept their follow-up appointments and, alarmingly, around 41% were lost to follow-up (Figure 2).

Post-intervention results

**Figure 3:**
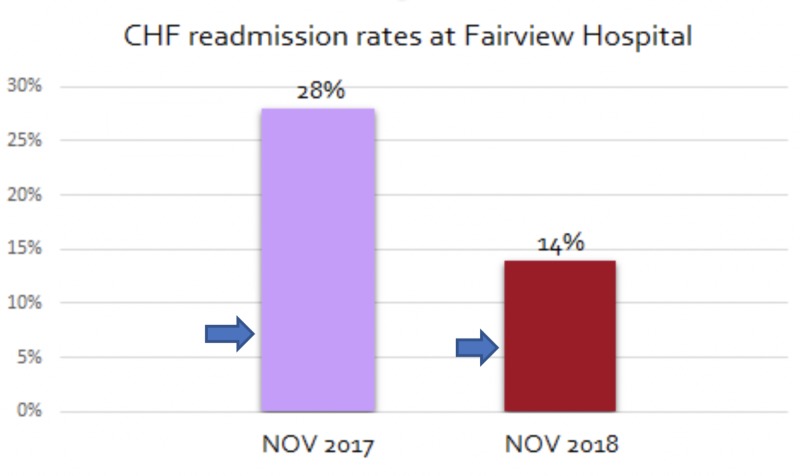
Comparison of readmission rates between the control and intervention groups NOV 2017 represents the control group and NOV 2018 represents the intervention group CHF: congestive heart failure

After the implementation of our project, around 60% of all patients who were discharged had a scheduled follow-up appointment with a PCP or cardiologist within the next two weeks. Also, about 56% of all discharged patients kept their follow-up appointments. More importantly, as a result of our interventions, we were able to reduce the 30-day all-cause readmission rates for our HF patients to 14%, which was a 50% reduction from the previous rates (Figure 3).

## Discussion

HF is a complex entity with a chronic progressive course. Though several interventions have been shown to be effective in improving the post-discharge course, readmission rates for HF still remain an issue. Previous studies have shown that early post-discharge follow-up is effective in preventing readmissions post-hospitalization [9]. However, only 37% of patients who were admitted to our hospital had usually made it to their follow-up visit. Through simple interventions such as encouraging patient education, stressing on the importance of lifestyle management, educating about medication compliance, and involving our ancillary staff to ensure that the patients had follow-up appointments scheduled before discharge, we were able to decrease our readmission rate by 50%. Educating the patients about the importance of maintaining their follow-up appointments was also critical in improving the number of patients who made it to their follow-up visits.

Patient education is an integral part of preventing readmissions. Education must start during the initial hospitalization and must continue during follow-up visits. Every future interaction with the patient should involve assessment and targeted education. Specific questions related to diet, medication compliance, and water intake should be asked. Such focused interaction will help in addressing knowledge gaps and in the reinforcement of previously discussed information. Studies have shown that education and reinforcement are pivotal in reducing HF readmissions [10].

Early follow-up visits with a cardiologist or PCP within seven days of discharge after an HF hospitalization have also been shown to help reduce the 30-day readmission rate [9]. As a part of our project, we were able to increase the number of patients who had a follow-up appointment scheduled with their PCP or cardiologist before being discharged from the hospital, and this translated into an increase in the number of people who made it to the follow-up appointments. Interventions such as weekly or biweekly phone calls, telemonitoring, and home visits can also be used to increase follow-up visits and thereby decrease readmission rates [10].

Ensuring a higher ratio of nursing staff has also been shown to be effective in reducing readmission rates [11]. This might have indirectly helped us in achieving our target as having adequate support from nursing staff to educate the patient was pivotal to our project. Optimizing medical therapy is also important to improve outcomes and reduce hospitalization for patients with HF. Beta-blockers, angiotensin-converting enzyme inhibitors or angiotensin receptor blockers, and spironolactone have been shown to have benefits and decrease readmissions in patients with HF with reduced ejection fraction [12-14]. Spironolactone has also been shown to reduce hospitalizations in patients with HF with preserved ejection fraction [15]. Compared to furosemide, patients discharged on torsemide were less likely to be readmitted for HF [16]. Thus, ensuring that patients are on the appropriate medication regimen during every hospital admission can help in preventing future hospitalizations.

On the other hand, whether the 30-day readmission rate is an appropriate metric to determine the quality of care provided is debatable. There is evidence to suggest that other factors such as socioeconomic status, patient factors, and community factors also play a role in this [5]. Also, exerting financial penalties on safety-net hospitals might adversely affect their ability to provide care for the vast majority of patients who depend on them [17]. Studies have also demonstrated an increase in 30-day and 1-year mortality for HF patients after the initiation of HRRP [7,18].

Thus, while it is important to introduce changes to reduce the 30-day readmission rates for HF patients, every effort must be taken to ensure that these do not adversely affect patient outcomes. More studies are required in this area to assess the short-term and long-term impact of such interventions and to examine if they can positively reduce the impact of HF on the patient and the society. The limitations of our study include the small sample size, short duration, and the lack of long-term follow-up. Also, our project was a single-center study and hence we cannot completely exclude the presence of other confounding factors.

Our findings imply that we can improve the readmission rates of HF hospitalizations with simple targeted interventions. Though some readmissions might not be preventable, we would like to emphasize that if adopted in a structured, enterprise-wide manner, these interventions can be as important as medications in improving the care of HF patients.

## Conclusions

Targeted interventions such as patient education and ensuring scheduled follow-up appointments before discharge are simple but effective tools that can help in reducing readmission rates for HF hospitalizations. Since HF readmissions exert a significant burden on the patients and society, such changes can help in improving patient care and reducing the pressure on the healthcare economy.
